# Lipid degradation promotes prostate cancer cell survival

**DOI:** 10.18632/oncotarget.16123

**Published:** 2017-03-11

**Authors:** Harri M Itkonen, Michael Brown, Alfonso Urbanucci, Gregory Tredwell, Chung Ho Lau, Stefan Barfeld, Claire Hart, Ingrid J. Guldvik, Mandeep Takhar, Hannelore V. Heemers, Nicholas Erho, Katarzyna Bloch, Elai Davicioni, Rita Derua, Etienne Waelkens, James L. Mohler, Noel Clarke, Johan V. Swinnen, Hector C. Keun, Ole P. Rekvig, Ian G. Mills

**Affiliations:** ^1^ Prostate Cancer Research Group, Centre for Molecular Medicine Norway, University of Oslo, Oslo, Norway; ^2^ Genito Urinary Cancer Research Group, Institute of Cancer Sciences, University of Manchester, Manchester, United Kingdom; ^3^ Department of Surgery and Cancer, Imperial College London, London, United Kingdom; ^4^ GenomeDx Biosciences, Vancouver, British Columbia, Canada; ^5^ Department of Oncology, Laboratory of Lipid Metabolism and Cancer, LKI Leuven Cancer Institute, KU Leuven-University of Leuven, Leuven, Belgium; ^6^ Department of Cellular and Molecular Medicine, Laboratory of Protein Phosphorylation and Proteomics, KU Leuven-University of Leuven, Leuven, Belgium; ^7^ Department of Cancer Biology, Cleveland Clinic, Cleveland, Ohio, USA; ^8^ Department of Urology, Cleveland Clinic, Cleveland, Ohio, USA; ^9^ Department of Hematology/Medical Oncology, Cleveland Clinic, Cleveland, Ohio, USA; ^10^ Department of Urology, Roswell Park Cancer Institute, Buffalo, New York, USA; ^11^ PCUK/Movember Centre of Excellence for Prostate Cancer Research, CRUK Manchester Institute for Cancer Research, University of Manchester, Manchester, UK; ^12^ Department of Urology, The Christie NHS Foundation Trust, Manchester, UK; ^13^ Department of Medical Biology, University of Tromso, Tromso, Norway; ^14^ Department of Molecular Oncology, Institute for Cancer Research and Oslo University Hospital, Oslo, Norway; ^15^ PCUK/Movember Centre of Excellence for Prostate Cancer Research, Centre for Cancer Research and Cell Biology (CCRCB), Queen's University Belfast, Belfast, UK

**Keywords:** androgen receptor, lipid degradation, metabolism, ECI2, cell cycle

## Abstract

Prostate cancer is the most common male cancer and androgen receptor (AR) is the major driver of the disease. Here we show that Enoyl-CoA delta isomerase 2 (ECI2) is a novel AR-target that promotes prostate cancer cell survival. Increased ECI2 expression predicts mortality in prostate cancer patients (*p* = 0.0086). *ECI2* encodes for an enzyme involved in lipid metabolism, and we use multiple metabolite profiling platforms and RNA-seq to show that inhibition of ECI2 expression leads to decreased glucose utilization, accumulation of fatty acids and down-regulation of cell cycle related genes. In normal cells, decrease in fatty acid degradation is compensated by increased consumption of glucose, and here we demonstrate that prostate cancer cells are not able to respond to decreased fatty acid degradation. Instead, prostate cancer cells activate incomplete autophagy, which is followed by activation of the cell death response. Finally, we identified a clinically approved compound, perhexiline, which inhibits fatty acid degradation, and replicates the major findings for ECI2 knockdown. This work shows that prostate cancer cells require lipid degradation for survival and identifies a small molecule inhibitor with therapeutic potential.

## INTRODUCTION

Prostate cancer is the most common cancer in men and targeting androgen receptor (AR) is an effective treatment. In the clinical setting, inhibition of AR activity frequently leads to the development of castration-resistant prostate cancer, a condition with no curative treatment options [1, 2]. AR signaling remains active in castration-resistant disease, and a recent study used chromatin immunoprecipitation (ChIP) coupled to sequencing (seq), to identify a gene signature of putative AR-target genes in this lethal form of cancer [3]. Among this signature was a poorly characterized enzyme, Enoyl-CoA delta isomerase 2 (ECI2). ECI2 has been purified, and using enzymatic assays, was shown to catalyze isomerization of lipids [4]. Lipid metabolism is of high interest in prostate cancer, since it offers a route to intra-tumoral steroid-hormone synthesis after or during androgen deprivation therapy [5]. The importance of steroidogenesis is further underlined by the anti-androgen abiraterone, which targets steroid metabolism and offers clinical benefit for castration-resistant prostate cancer patients [6].

The metabolic status of the normal prostate is different from the rest of the body due to the incomplete TCA cycle and secretion of citrate, a TCA metabolite, important for the viability of sperm [7]. In the early stages of prostate cancer, citrate secretion decreases, accompanied by a decrease in aerobic glycolysis, which ‘masks’ tumors from the conventional tracers of proliferating cells, such as glucose-PET [8, 9]. Lipid metabolizing enzymes are up-regulated in the early phase of prostate cancer and remain elevated throughout the disease progression, which suggests an active role in the disease [9]. In agreement with this, statin intake to lower blood cholesterol levels is associated with prostate cancer prevention [10]. The importance of lipid metabolism is exemplified further by the lipid producing enzyme, fatty acid synthase, which is over-expressed during prostate cancer progression and can be targeted with Orlistat, a drug approved for the treatment of obesity [11, 12].

Since AR activity and lipid metabolism appear important in prostate cancer, here we investigate *ECI2*, an AR-target gene. We show that ECI2 is over-expressed in prostate cancer patient samples in mRNA and protein levels, and that increased expression predicts poor outcome. Knockdown of ECI2 had minimal effect on gene expression in a cell line representing normal prostate epithelium but resulted in down-regulation of cell cycle-associated genes in prostate cancer cells. The difference in gene expression translated to growth inhibition of prostate cancer cells, but had only modest effect on a cell line representing normal prostate epithelium. Integration of the RNA-seq data with metabolomics data revealed that inhibition of ECI2 expression led to acute metabolic stress in prostate cancer cells.

## RESULTS

### ECI2 is over-expressed in clinical prostate cancer

**Figure 1:**
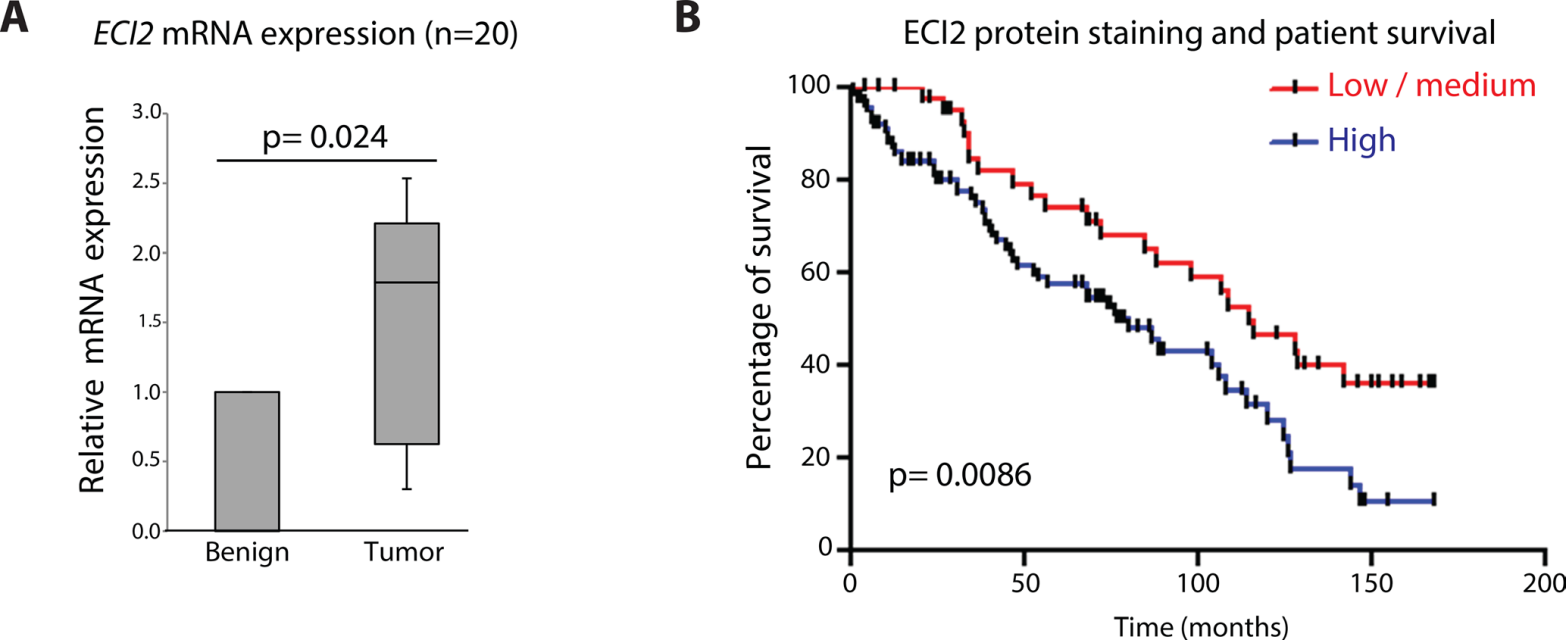
Enoyl-CoA delta isomerase 2 (ECI2) is over-expressed in prostate cancer (**A**) ECI2 expression was evaluated in prostate cancer tissue samples. The data shown represents matched normal epithelium and adenocarcinoma from 20 radical prostatectomy specimens. Relative expression of the different transcripts were calculated using the comparative CT method, where the matched benign tissue of the same patient were set to 1 and normalized to the geometric mean CT value of GAPDH, TBP and 18s. Wilcoxon matched-pairs signed rank test was used to test for significance in the differential expression of ECI2 between the matched benign and cancer tissue. (**B**) Kaplan Meier curves for the low/medium group versus the high ECI2 expressing group. We evaluated whether ECI2 expression levels are associated with survival in prostate cancer patients. The difference in overall survival between the low/medium expressing group and high expressing group was 77 months vs 115 months, *p* = 0.0086. Here stating that an overview of the clinical cohorts use in Figures 1A and 1B and the statistical analysis are to be found in Supplementary Tables 2, 4 and 5.

*ECI2* was identified as a putative AR target gene in castration-resistant prostate cancer tissue samples using ChIP-seq technology [3]. As the first step, we evaluated *ECI2* expression in matched benign and prostate cancer patient tissue samples, and observed a 2-fold increased expression of *ECI2* mRNA (*p* = 0.024, Figure 1A). Encouraged by this result, we evaluated ECI2 protein level expression using immunohistochemistry, and found out that elevated ECI2 protein predicted mortality (*p* = 0.0086, Figure 1B, see also Supplementary Figure 1).

**Figure 2:**
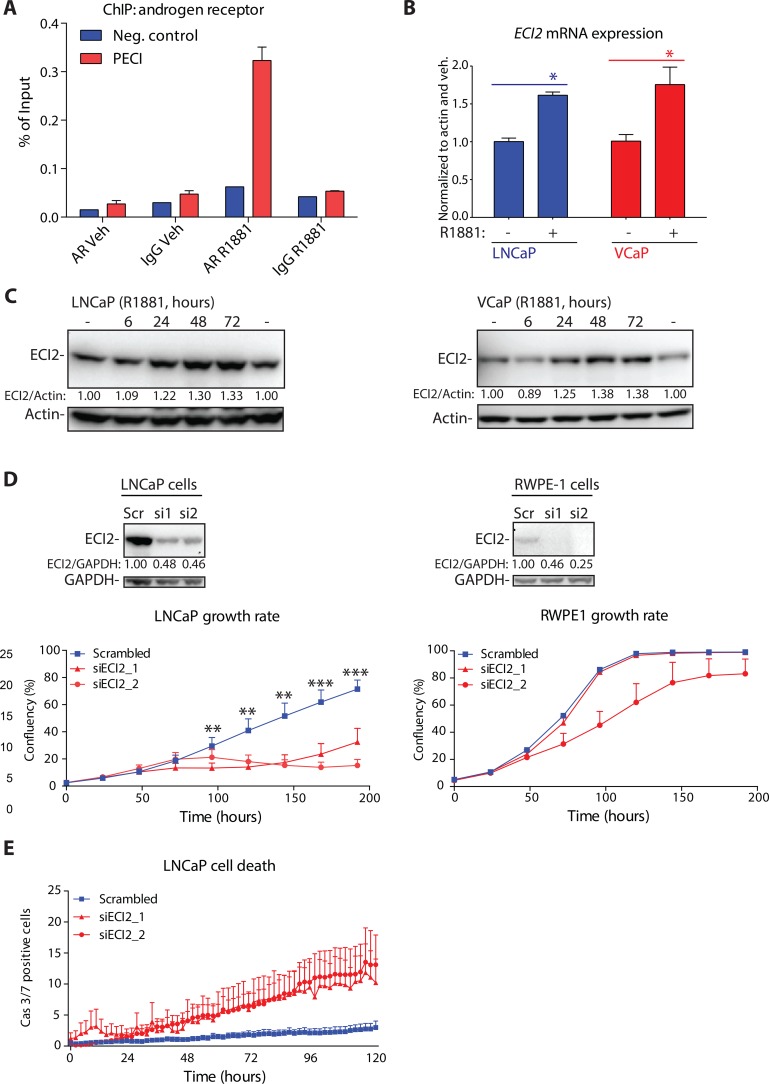
Androgen receptor (AR) regulates Enoyl-CoA delta isomerase 2 (ECI2) expression (**A**) Chromatin immunoprecipitation (ChIP) of androgen receptor (AR) in VCaP cells. Cells were deprived of androgens for 3 days and treated either with 1nM R1881 or vehicle, as indicated. The putative AR binding site for ECI2 was identified from a published AR ChIP-seq data set [3]. The data shown is representative of two biological replicates. (**B**) LNCaP and VCaP cells were treated as in A. Total mRNA was isolated at 12 hours and the expression of *ECI2* and *actin* was evaluated using RT-qPCR. The data shown are an average of three independent experiments with SEM, and significance was evaluated using paired samples Student's *t-test*, *< 0.05. (**C**) LNCaP and VCaP cells were deprived of androgens for 3 days, treated either with 1nM R1881 or vehicle (−) and protein lysates were collected. Densitometry was used to evaluate ECI2 levels. **(D**) Western blot was used to confirm ECI2 knockdown in LNCaP and RWPE-1 cells after 96 hours. Densitometry was used to evaluate ECI2 levels. The growth rate of cells was evaluated with life-cell imaging. The data shown are an average of three independent biological replicates with SEM. The significance was evaluated using paired samples Student's *t-test*, **< 0.01, ***< 0.001. (**E**) Cell death activation after ECI2 knockdown. LNCaP cells were reverse-transfected and allowed to attach for 24 hours. At this point, a dye detecting caspase 3/7 activation was added and cumulative activation of caspase 3/7 was followed in real-time using life-cell imaging. Data shown is an average of three biological replicates with SEM.

Since ECI2 was over-expressed in prostate cancer patient samples, we moved on to assess AR-dependent regulation of this gene. We re-analyzed AR ChIP-seq data from human tissue samples [3], and putative AR-binding site in castration-resistant prostate cancer patient samples was found inside the *ECI2* gene body (chromosome coordinates in Human Genome 18: chr6:4,075,826-4,076,114). In order to confirm these data, we designed primers against this site, and assessed potential AR binding using ChIP-qPCR. Androgen-stimulation resulted in 6-fold increased AR binding to this site, once compared to vehicle and an IgG antibody control (Figure 2A). We next confirmed that androgen stimulation increases ECI2 expression at the mRNA and protein levels in LNCaP and VCaP cells (Figure 2B and 2C). Information on the primers and probes used in this study for ChIP-qPCR and RT-PCR are to be found in Supplementary Table 3 and more detailed methodology is provided in Supplementary Materials.

Since ECI2 was over-expressed in prostate cancer patient samples, we tested whether the enzyme is important for prostate cancer cell growth by following cell proliferation using life-cell imaging. ECI2 knockdown strongly inhibited proliferation of LNCaP prostate cancer cell line but had only modest effects on RWPE-1, a cell line derived from normal prostate epithelium (Figure 2D). In addition, knockdown of ECI2 activated cell death response in LNCaP cells (Figure 2E).

These data suggest that ECI2 has a role in promoting the proliferation of prostate cancer cells.

### Metabolomic profiling after ECI2 knockdown revealed profound changes in lipid composition

**Figure 3:**
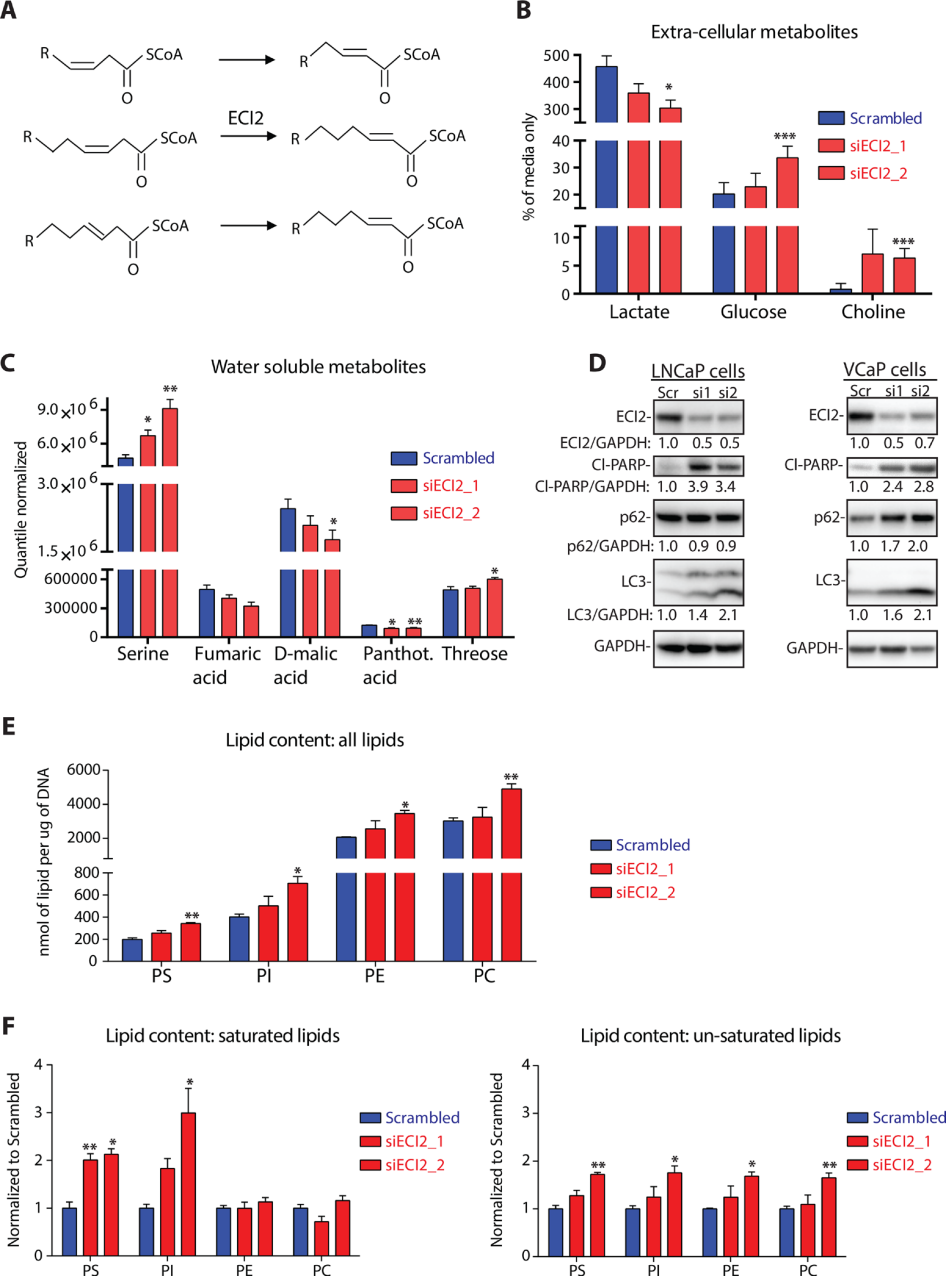
Metabolomic profiling after ECI2 knockdown in LNCaP cells (**A**) Reaction catalyzed by Enoyl-CoA Delta Isomerase 2 (ECI2). (**B**) The level of extra-cellular lactate, glucose and choline were determined using nuclear magnetic resonance after 72 hours of ECI2 knockdown. The data are presented as % of complete media without cells and are an average (with SEM) of five biological replicates. The significance was evaluated using paired samples Student's *t-test*, *< 0.05, ***< 0.001. (**C**) The levels of water-soluble intra-cellular metabolites were determined using mass-spectrometry after 72 hours of ECI2 knockdown. Only metabolites that were affected significantly by at least one siRNA are shown. Data shown are an average (with SEM) of five biological replicates. The significance was evaluated using paired samples Student's *t-test*, *< 0.05, **< 0.01. (**D**) ECI2 knockdown in LNCaP (for 72 hours) and VCaP cells (for 96 hours). The data shown are representative of at least three biological replicates for LNCaP cells and two replicates for VCaP cells. Densitometry was used to evaluate signal intensity. (**E**) The levels of intra-cellular phosphatidylserine (PS), phosphatidylcholine (PC), phosphatidylethanolamine (PE) and phosphatidylinositol (PI) were determined using mass-spectrometry after 72 hours of ECI2 knockdown. Identification of lipid composition is provided in Supplementary Figure 2. Data shown are an average (with SEM) of three biological replicates. The significance was evaluated using paired samples Student's *t-test*, *< 0.05, **< 0.01. (**F**) The levels of saturated and un-saturated lipids were determined using mass-spectrometry after 72 hours of ECI2 knockdown (same data-set as presented in Figure 3E). Scrambled samples were set to 1 for all four lipid classes, and siECI2 samples were normalized to this. Data shown are an average (with SEM) of three biological replicates. The significance was evaluated using paired samples Student's *t-test*, *< 0.05, **< 0.01.

ECI2 has been reported to function in lipid processing and the enzyme has been shown to isomerize 3-cis-octenoyl-CoA to 2-trans-octenoyl-CoA [4] (Figure 3A). This isomerization reaction is important for the subsequent degradation of unsaturated lipids [4]. Degradation maintains correct lipid homeostasis, supports citric acid cycle by production of acetyl-CoA and replenishes NADH and FADH pools.

ECI2 knockdown decreased glucose consumption and lactate production, as measured from cell culture media (Figure 3B). In order to get a clearer picture of the prostate cancer cell metabolome after ECI2 knockdown, we used mass spectrometry-based untargeted metabolite profiling of intra-cellular metabolites. This approach revealed decrease in TCA cycle metabolites fumarate and malate, while serine accumulated (Figure 3C).

These changes suggest that ECI2 knockdown imposes an acute metabolic stress on cells. Therefore we would expect stress-resistance promoting pathways, such as autophagy, to be up-regulated [13]. ECI2 knockdown induced prominent accumulation of the canonical autophagy marker LC3 [14], in two prostate cancer cell lines, LNCaP and VCaP (Figure 3D). However, p62, an adaptor protein for autophagy [14], was not degraded, which suggests incomplete autophagy and sustained stress. Indeed, prostate cancer cells were unable to respond to ECI2 knockdown induced metabolic stress, which resulted in cell death activation (PARP cleavage, Figure 3D).

The major task for ECI2 enzyme is believed to be lipid processing [4], and we observed choline accumulation in culture media after ECI2 knockdown (Figure 3B). Based on these facts, we performed targeted phospholipidomic profiling of LNCaP prostate cancer cells after ECI2 knockdown, which revealed accumulation of all major classes of phospholipids, phosphatidylserine (PS), phosphatidylcholine (PC), phosphatidylethanolamine (PE) and phosphatidylinositol (PI) (Figure 3E and Supplementary Figure 2). ECI2 knockdown had prominent effect on the saturated lipids of PS and PI (Figure 3F). However, this lipid pool represents a minor fraction of total cellular lipids, and in this context it is important to point out that the most abundant lipids in scrambled condition were profoundly increased upon ECI2 knockdown (Supplementary Figure 2). For example lipids with single double-bond, especially 36:1, were prominently increased in all four lipid classes.

We next used Oil Red O staining to confirm a statistically significant increase of lipids (Supplementary Figure 3A). Based on Oil Red O staining, we noted possible accumulation of lipid droplets, and this was confirmed with AdipoRed stain (Supplementary Figure 3B).

We have so far shown that ECI2 knockdown inhibits cancer cell proliferation, which is associated with decreased glucose consumption and lipid accumulation. This caused acute stress and cell death activation, but exact mechanism(s) remain unclear.

### ECI2 knockdown represses cell cycle associated genes

**Figure 4:**
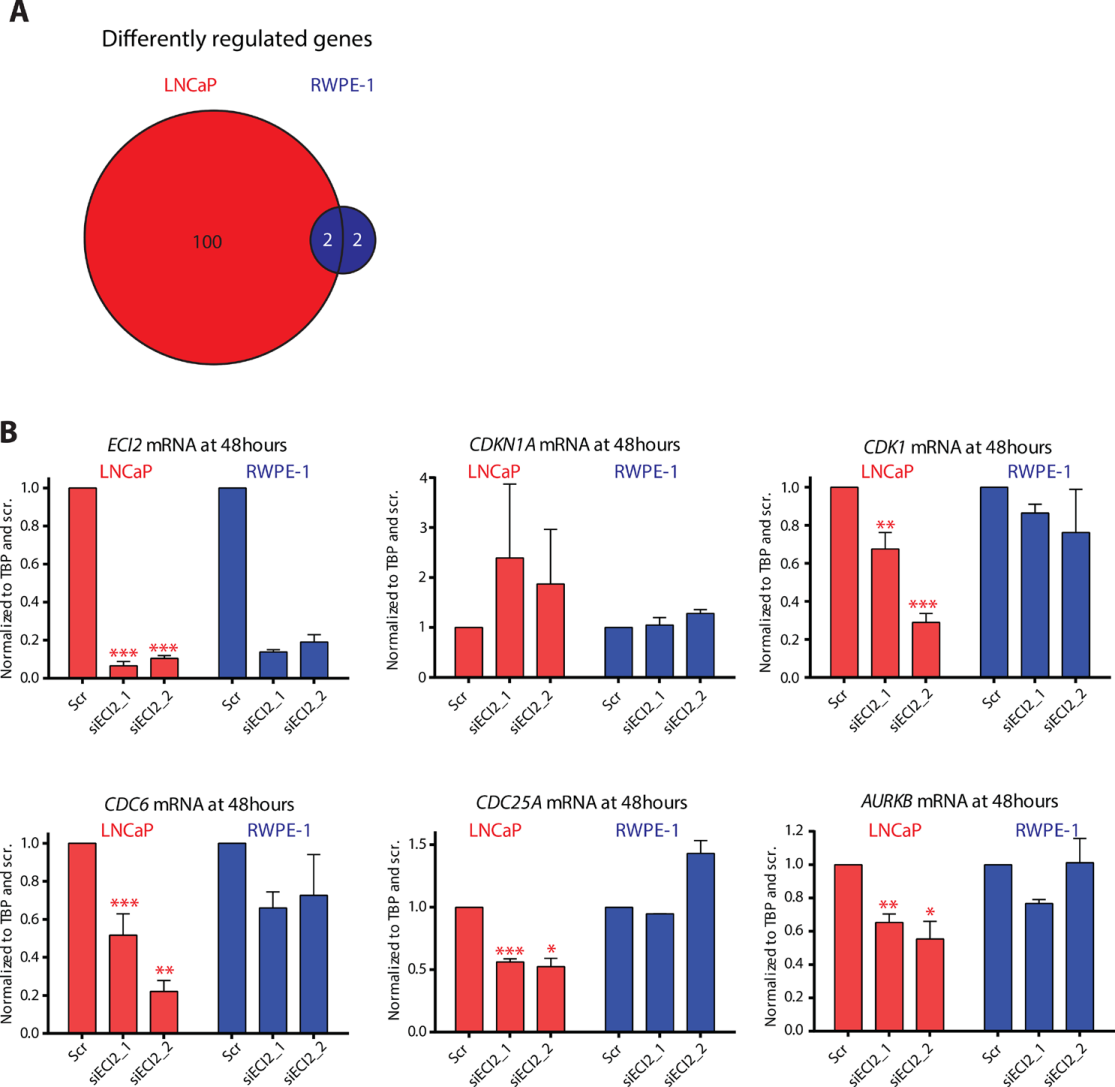
RNA-seq after ECI2 knockdown in LNCaP and RWPE-1 cells The expression of ECI2 was reduced by treating LNCaP and RWPE-1 cells for 48 hours with siRNA and RNA was collected and used for RNA-seq. (**A**) Venn diagram shows the number of genes that were differentially regulated by both siRNAs in either LNCaP or RWPE-1 cells, and regulated differentially between the two cell lines. (**B**) Validation of the RNA-seq data using RT-qPCR. The data shown are an average of at least two biological replicates for both RNA-seq and validation, and the significance was evaluated using paired samples Student's *t-test*, *< 0.05, **< 0.01, ***< 0.001.

In order to better understand metabolomics data, we performed RNA-sequencing after ECI2 knockdown in the LNCaP prostate cancer cell line and RWPE-1 cell line, which represents normal prostate epithelial cells. In order to set stringent criteria to establish ECI2-dependent transcripts, we performed RNA-seq on two biological replicates and selected only the transcripts that were affected by both of the two siRNAs targeting ECI2. A summary of the differentially expressed genes in each cell-line and in each biological replicate is provided in Supplementary Table 6. Based on this approach, the expression of 4 genes was altered in RWPE-1 cells and the expression of 102 genes was altered in LNCaP cells after ECI2 knockdown at the selected 48 hour time point (Figure 4A). We first inspected the individual genes differently regulated in RWPE-1 and LNCaP cells. Interestingly, RWPE-1 but not LNCaP cells, increased the expression of stress-resistance factor *HSPB1* (heat shock protein family B (small) member 1) (Supplementary Table 1), which is known to protect cells from stress-induced apoptosis [15]. On the other hand, we found a number of genes known to be over-expressed in prostate cancer and promote prostate cancer cell survival, which were profoundly down-regulated only in LNCaP cells (Supplementary Table 1). Two examples are *MELK* (Maternal Embryonic Leucine Zipper Kinase) [16] and *WASF3* (WAS Protein Family Member 3) [17, 18]. These data support the hypothesis that ECI2 has more important function(s) in prostate cancer cells.

In order to understand potential transcriptional programs of how prostate cancer cells respond to knockdown of ECI2, we next focused on the genes that were up-regulated after ECI2 knockdown only in LNCaP cells and performed pathway enrichment analysis using the Database for Annotation, Visualization and Integrated Discovery (DAVID) [19, 20]. This analysis highlighted two pathways, ‘p53 signaling pathway’ and ‘Chronic myeloid leukemia’, with one common gene, *CDKN1A* (Supplementary Table 1). *CDKN1A* functions as a cyclin-dependent kinase inhibitor [21]. We next performed the pathway enrichment analysis with the genes that were down-regulated in LNCaP cells after ECI2 knockdown. Four pathways were highlighted, and the most significant one was ‘Cell cycle’, through decreased expression of *CDK1, CDC6, MAD2L1, CCND3, CDKN2C, CDC25A, WEE1* and *CDC25B* (Supplementary Table 1). We used RT-qPCR to confirm the statistically significant differential expression of *CDKN1A, CDK1, CDC6, CDC25A* and *AURKB* in LNCaP and RWPE-1 cells after ECI2 knockdown (Figure 4B). ECI2 knockdown also statistically significantly decreased the expression of CDC6 in VCaP cells (Supplementary Figure 4).

RNA-seq and subsequent validation revealed that *CDC6* is one of the most prominently down-regulated genes in both prostate cancer cell lines. Potential correlation between *CDC6* expression and prostate cancer aggressiveness has not been previously evaluated, but this protein is normally found in proliferating cells [22]. We therefore evaluated the correlation between elevated *CDC6* expression and metastasis in a cohort of high-risk prostate cancer patients [23]. In this cohort, increased *CDC6* expression predicted metastasis (*p* = 7.41e-04), and was also prominently associated with prostate cancer-specific mortality (*p* = 1.09e-02, Supplementary Figure 5).

So far, we have identified a gene, *ECI2*, for which knockdown inhibited prostate cancer cell proliferation and in addition suppressed expression of *CDC6*. Elevated expression of *CDC6* is associated with poor patient outcome. Based on these data, ECI2, or more generally, lipid degradation, might represent a novel drug target to limit prostate cancer cell proliferation.

### Targeting lipid degradation inhibits prostate cancer cell proliferation

There are no drugs directly inhibiting ECI2 activity but small molecule inhibitors targeting lipid degradation are available. Some of these compounds, including perhexiline, are available clinically [24]. Perhexiline is used to inhibit degradation of lipids for energy production, and thereby to promote more oxygen-efficient utilization of glucose as an energy source in chronic ischemic cardiomyopathy [25]. Based on our metabolomics and RNA-seq data, prostate cancer cells are unable to increase glucose utilization once lipid degradation is inhibited. Instead, an acute metabolic stress is induced, which culminates in cell death response activation (Figure 3D). We hypothesized that perhexiline might be able to cause similar response in prostate cancer cells.

**Figure 5:**
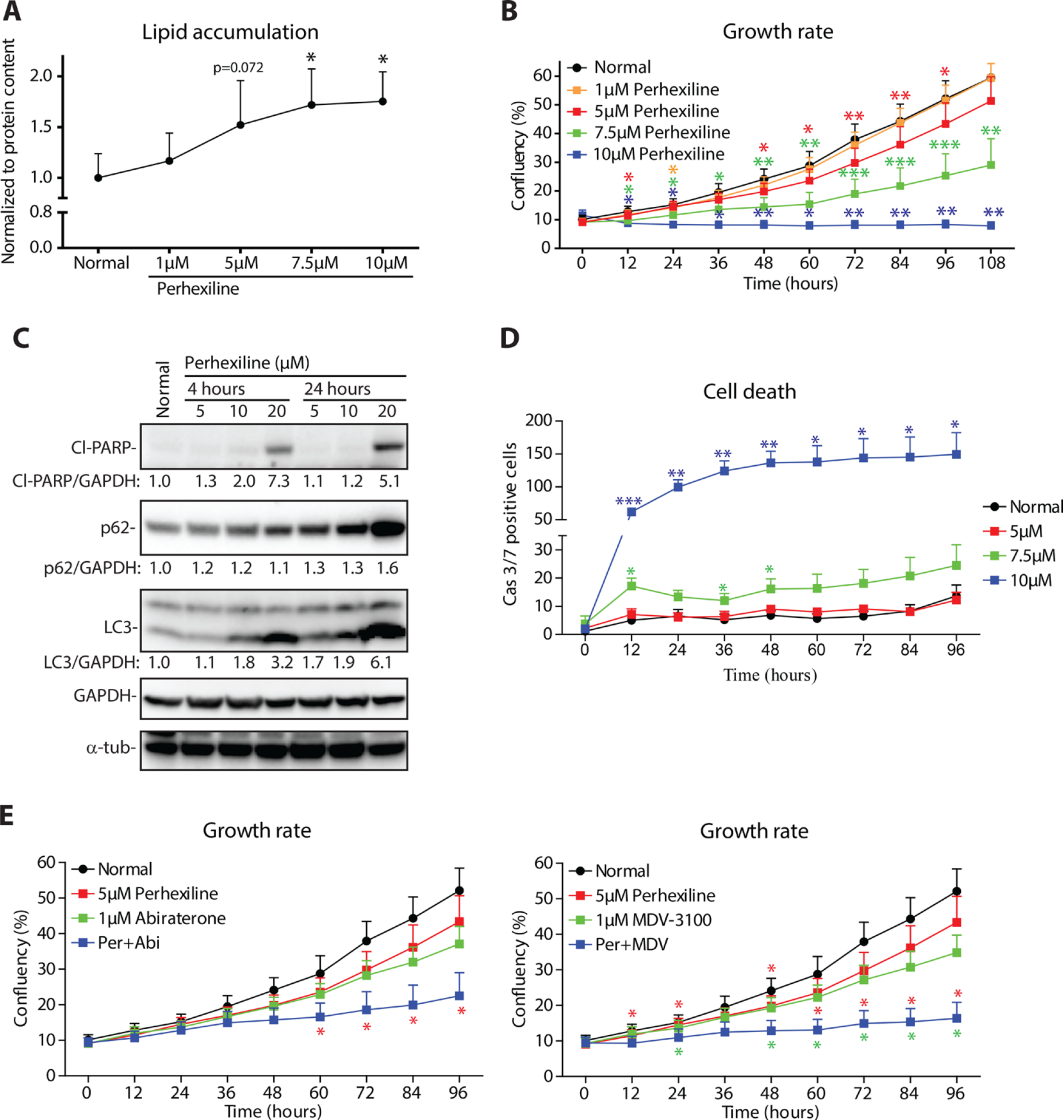
Lipid degradation inhibitor perhexiline activates cell death in prostate cancer cells (**A**) Oil red O staining of LNCaP cells after 24 hours of perhexiline treatment. Data shown are an average (with SEM) of five biological replicates. The significance was evaluated using paired samples Student's *t-test*, *< 0.05. (**B**) LNCaP cells were treated with increasing doses of perhexiline and growth rate was followed using life-cell imaging. Data shown is an average of four biological replicates with SEM. Significance was evaluated using paired samples Student's *t-test*, *< 0.05, **< 0.01, ***< 0.001. (**C**) LNCaP cells were treated with perhexiline for 4 hours or 24 hours, and protein lysates were collected for western blotting. The data shown are representative of three biological replicates. Densitometry was used to evaluate signal intensity. (**D**) LNCaP cells were treated with perhexiline as indicated and cell death activation was evaluated using life-cell imaging detecting activation of caspases 3 and 7. Data shown is an average of four biological replicates with SEM. Significance was evaluated with paired samples Student's *t-test*, *< 0.05, **< 0.01. (**E**) LNCaP cells were treated with perhexiline alone or in combination with androgen deprivation therapy (either Abiraterone or MDV-3100) and growth rate was followed using life-cell imaging. Data shown are an average of four biological replicates with SEM. Significance was evaluated using paired samples Student's *t-test*, *< 0.05. Red stars indicate comparison between perhexiline and combinatorial treatment, while green stars indicate comparison between androgen deprivation and combinatorial treatment.

We first confirmed that perhexiline treatment leads to dose-dependent lipid accumulation, similar to that observed upon ECI2 knockdown (Figure 5A). This lipid accumulation was mirrored by concomitant decrease in prostate cancer cell proliferation (Figure 5B). Similar to ECI2 knockdown, prostate cancer cells responded by activation of incomplete autophagy, as measured by prominent accumulation of autophagy markers LC3 and adaptor protein p62 [14] (Figure 5C). Prostate cancer cells were unable to respond to perhexiline-induced metabolic stress, and higher doses induced cell-death, as measured by PARP cleavage and activation of caspases 3/7 (Figure 5C and 5D). In addition, this compound let to prominent down-regulation of CDC6 (Supplementary Figure 6).

These data suggest that perhexiline could have potential in limiting the proliferation of prostate cancer cells, but one of the challenges in utilizing perhexiline in clinical setting are ‘poor-metabolizers’, patients who have altered activity of CYP2D6 (cytochrome P450 family 2 subfamily D member 6) [26]. This problem can be overcome by dose-reduction and/or genetic testing [27]. The dose-limitation challenge motivated us to evaluate whether combinatorial treatment with perhexiline and anti-androgens would allow dose reduction. For these experiments, we selected a low dose of perhexiline (5 μM), which on its own led to prominent increase in intracellular lipid content but only modestly inhibited proliferation (Figure 5A and 5B). Interestingly, combinatorial treatment of LNCaP cells with perhexiline and either Abiraterone or Enzalutamide (MDV-3100) almost completely blocked proliferation (Figure 5E).

Taken together, these data suggest that a novel AR target gene, *ECI2*, supports aberrant metabolic homeostasis of prostate cancer cells. Inhibition of lipid degradation either by knocking down this enzyme or by a small molecule inhibitor leads to metabolic stress and activation of the cell death response.

## DISCUSSION

Prostate cancer is the most common cancer in men, and increased AR activity is associated with prostate cancer development and progression. AR-driven complex transcriptional programs have previously been used as a starting point to identify prostate cancer-specific metabolic vulnerabilities [28, 29]. In this study, we showed that ECI2, an enzyme involved in degradation of unsaturated lipids, is a direct AR-target and over-expressed in clinical prostate cancer (Figures 1 and 2). Both ECI2 and CDC6 can be induced by AR (this paper and Mallik *& al*. 2008 [30], respectively). Inhibition of ECI2 expression decreased the growth rate and activated cell death response in prostate cancer cells (Figures 2D and 3D).

Proliferating cancer cells have increased need for lipids [31]. Interestingly, here we observed that inhibition of ECI2 expression and perhexiline treatment led to prominent accumulation of lipids into cells, which did not promote cancer cell proliferation. Normal prostate cells have an incomplete TCA cycle and therefore cannot rely on typical energy producing pathways [9, 11]. This metabolic adaptation has been shown to sensitize prostate cancer cells to compounds inhibiting mitochondrial activity, and specifically sensitize the cancer cells to other treatments [32]. Here we propose that this metabolic adaptation forces prostate cancer cells to derive a significant fraction of their energy through alternative pathways, such as lipid degradation. In agreement with this model, inhibition of lipid degradation decreased glucose utilization and led to accumulation of starvation /autophagy markers (Figure 3D and Figure 5C) [14]. Instead of successful, cell-survival promoting autophagy, inhibition of lipid degradation led to the activation of the cell death response in prostate cancer cells. Similar dependency on fatty acid oxidation was recently reported in MYC-overexpressing triple-negative breast cancer cells [33]. In breast cancer cells, perhexiline has been shown to inhibit HER3-signaling [34] and induce autophagy [35]. It is not known whether these effects are caused directly by the compound or are down-stream effects from inhibition of lipid metabolism, as it is well established that perhexiline affects lipid metabolism of cancer cells [36, 37]. Interestingly, a number of studies have reported that perhexiline is able to sensitize, and in some cases, reverse resistance, to clinically relevant drugs, including doxorubicin in breast cancer cells [38] and murine leukemia cell line [39] and cisplatin in neuroblastoma cells [40]. We are the first to show that perhexiline has prominent anti-tumor activity once combined with second-generation anti-androgens, Abiraterone and Enzalutamide (Figure 5E). However, because cancer metabolism is influenced by the tumour microenvironment and can be compartmentalized between different cell populations [41–43], it will be important in the future to extend studies of ECI2 function and perhexiline response to more patient-derived pre-clinical models (eg. Explants or PDX) as a stepping stone to clinical trials.

Perhexiline can cause severe toxic side-effects, including neuro- and hepatotoxicity, in a sub-set of patients [27]. The majority of the side-effects are caused by the complex pharmacokinetic profile of this compound, which results in bio-accumulation in small number of patients and sub-sequent toxicicity [26, 27]. This toxicity can be minimized by sustained monitoring of perhexiline plasma levels and adjusting the drug-dosing to result in blood levels below 600 ng/ml [44–46]. It is noteworthy that the anti-tumor activity of perhexiline has been demonstrated in mouse models of several cancers, including breast cancer [34], neuroblastoma [40] and chronic lymphocytic leukemia [36] with no obvious toxicity.

Normal cells are able to compensate for decreased lipid degradation through increased glucose consumption, and therefore inhibition of lipid utilization is not toxic [47]. In fact, certain tissues essentially require glucose as an energy source, and in certain instances inhibition of lipid oxidation is beneficial. This is the case in chronic ischemic cardiomyopathy, a condition with limited oxygen supply to heart tissue [25].

In summary, we propose that prostate cancer cells essentially require lipid degradation for energy production and to sustain proliferation. Failure to adapt to inhibition of lipid degradation leads to profound changes in total metabolome and eventually activation of the cell death response in prostate cancer cells.

Targeting cancer-cell specific metabolic vulnerabilities with approved compounds is an exciting approach, and should speed the development of new combinatorial therapies. Clinical trials using metabolic inhibitors in combination with established cancer-specific drugs are on-going, including compounds such as metformin (for details see: https://clinicaltrials.gov/), and scientific community will likely see more of those in the future. Our data provide evidence to combine anti-androgens with compounds targeting lipid metabolism.

## MATERIALS AND METHODS

### Evaluation of ECI2 expression in patient samples

RNA extracted from surgical specimens was obtained from Roswell Park Cancer Institute. These samples are covered by an approval (BDR 034313) from the Office of Research Subject Protection. The Salford prostate TMA consisting of diagnostic needle core biopsies from 144 patients attending the Urology clinic of Salford Royal NHS Foundation Trust was covered by MCRC Biobank Ethics 10_NOCL_02, Manchester, UK. Detailed protocols are provided in Supplementary Materials.

### Cell lines, maintenance and treatments

Cells were obtained from ATCC and maintained according to ATCC guidelines. To simulate androgen deprivation, cells were kept in phenol-red free media supplemented with charcoal-stripped serum. Knockdown of ECI2 was performed with RNAiMAX reagent (Thermo Fisher Scientific) according to manufacturer's instructions. ECI2 targeting siRNAs were obtained from Qiagen (SI04202282 and SI04201848). Perhexiline was purchased from Sigma (catalogue number: SML0120-10MG). More detailed protocols for cell lysate preparation, Oil Red O staining (obtained from Sigma, catalogue number: O1391-250ML) and Lipid Tox staining (obtained from Life Technologies, catalogue number: H34475) are available in Supplementary Materials.

### Analysis of viability, growth rate and caspase activity

Cells were plated on 384-well plates one day before treatment. Caspase 3/7 activation was detected using IncuCyte^®^ Caspase-3/7 Apoptosis Assay Reagent (EssenBiosciences, catalogue number 4440) and using the IncuCyte instrument (EssenBiosciences) according to manufacturer's instructions. Growth rates were determined using the IncuCyte instrument according to manufacturer's instructions. In brief: in order to determine growth rate of cells, cells were reverse-transfected into 384-well plate, and the plate was placed into IncuCyte instrument. Using this instrument, cells were imaged as indicated in the figures and confluency was determined using the built-in software that comes along with the instrument. In order to determine growth rate of cells after compound treatment, cells were plated one day before the treatments, and on the next day, cells were treated as indicated in the figure and growth rate was followed by life-cell imaging.

### Metabolomics, RNA-seq and mayo cohort analysis

Detailed protocols and analysis of Gas-chromatography mass spectrometry, ^1^H NMR spectroscopy of cell culture media, lipidomics and RNA-seq, ChIP-seq and analysis of Mayo cohort samples for met- and prostate cancer specific mortality-free survival are provided in Supplementary Materials. RNA-seq data has been made available: http://www.ncbi.nlm.nih.gov/geo/query/acc.cgi?token=wxydygwubzcxxax&acc=GSE75035 for editor/reviewers, and will be made publicly available once the manuscript has been accepted. The processed files are compressed folders containing multiple output files from CuffDiff runs estimating differentially expressed transcripts between the indicated ECI2 siRNA treated cells versus cells treated with Scrambled siRNAs (see Trapnell *et al*., 2012 for more info [48]).

## SUPPLEMENTARY MATERIALS FIGURES AND TABLES



## Abbreviations

AR –: Androgen receptor
AURKB –: Aurora kinase B
CCND3 –: Cyclin D3
CDC6 –: Cell division cycle 6
CDC25A –: Cell division cycle 25A
CDC25B –: Cell division cycle 25B
CDK1 –: Cyclin dependent kinase 1
CDKN1A –: Cyclin dependent kinase inhibitor 1A
CDKN2C –: Cyclin-dependent kinase inhibitor 2C
ChIP –: Chromatin immunoprecipitation
ChIP-seq –: Chromatin immunoprecipitation coupled to sequencing
Cl-PARP –: Cleaved poly(ADP-ribose) polymerase
CYP2D6 –: Cytochrome P450 family 2 subfamily D member 6
ECI2 –: Enoyl-CoA delta isomerase 2
GAPDH –: Glyceraldehyde-3-phosphate dehydrogenase
LC3 –: Microtubule associated protein 1 light chain 3 alpha
MAD2L1 –: MAD2 mitotic arrest deficient-like 1
MELK –: Maternal Embryonic Leucine Zipper Kinase
p62 –: Sequestosome 1
TBP –: TATA-box binding protein
WASF3 –: WAS Protein Family Member 3
WEE1 –: WEE1 G2 checkpoint kinase.
