# Supplementary material for: Lipid degradation promotes prostate cancer cell survival
Source: Oncotarget. 2017 Mar 11;8(24):38264–75. doi: 10.18632/oncotarget.16123 (PMC5503531; doi:10.18632/oncotarget.16123)
Supplement: Supplementary file 1 [file oncotarget-08-38264-s001.pdf]

Lipid degradation promotes prostate cancer cell survival

Supplementary Materials

SUPPLEMENTARY METHODS

mRNA profiling of patient tissue

The RNA samples represent matched normal epithelium and adenocarcinoma (at least 70% neoplastic nuclei) from 20 radical prostatectomy specimens (for further information, see Supplementary Table 2). Tissue procurement has been described [1]. RNA isolation was performed using Trizol extraction. Two-hundred nanograms of RNA were reverse-transcribed using High-Capacity RNA-to-cDNA master mix (Applied Biosystems), prior to a target-specific pre-amplification step using TaqMan PreAmplification Master Mix (Applied Biosystems). The amplified cDNA was diluted 1:20 in 1x TE, and 2.5 uL were used per reaction, in a total of 10 uL per well. Quantitative real-time PCR was performed using TaqMan Gene Expression Assays (Applied Biosystems) and TaqMan Fast Advanced PCR Master Mix (Applied Biosystems) (Supplementary Table 3). PCR amplification was performed in duplicate series using the ABI 7900HT FAST Sequence Detection System (Applied Biosystems). The cycling conditions were 50°C for 2 minutes, 95°C for 10 minutes, followed by 40 cycles of 95°C for 15 seconds, and 60°C for 1 minute. Relative expression of the different transcripts were calculated using the comparative CT method, where the matched benign tissue of the same patient were set as 1 and normalized to the geometric mean CT value of GAPDH, TBP and 18s [2]. Wilcoxon matched-pairs signed rank test was used to test for significance in the differential expression of ECI2 between the matched benign and cancer tissue.

Immunohistochemistry

The Salford prostate TMA consisting of diagnostic needle core biopsies from 144 patients attending the Urology clinic of Salford Royal NHS Foundation Trust was covered by MCRC Biobank Ethics 10_NOCL_02, Manchester, UK. PCa tissue from diagnostic needle cores was identified and Gleason graded by a consultant histopathologist, with Gleason grading being confirmed by an independent consultant histopathologist upon presentation within the TMA block (tissue demographics are provided in Supplementary Table 4). ECI2 antibody validation and example of staining are provided in Supplementary Figure 1. The TMA was stained using the automated Ventana Benchmark Ultra Automated IHC/ISH Slide Staining System with manual application of 1:500 dilution ECI2 (Sigma HPA022130) and 1:50 dilution TSA Cyanine 5 antibodies (Tyramide Signal Amplification Cyanine 5 Open 3 Plex Kit; Perkin Elmer) prior to manual co-staining with 2.5 µg/ml pan-cytokeratin-PE (Sigma SAB4700668) and DAPI. Slides were mounted in Prolong Gold (Invitrogen). Automated image analysis was performed using Definiens Tissue Studio 3.0 (Definiens AG, München, Germany). Immunofluorescent sections were scanned using 3D Histech Mirax scanner (Carl Zeiss Microimaging) and imported into Definiens Tissue Studio (Definiens AG, Munich, Germany). Operator screening was used to provide quality controls for automated core detection and region of interest (ROI) identification. Epithelial cells were identified by pan-cytokeratin staining. ECI2 staining within epithelial cells was identified using an in house developed algorithm with intensity thresholds (pixel density) to classify cells as negative ($\leq$ 40), low ($\leq$ 100), intermediate ($\leq$ 180) or high expression. Prostate cancers were categorized as the highest threshold when $\geq$ 2% of cells expressed ECI2. Expression data were correlated with outcome data using Definiens Image Miner (Definiens AG, Munich, Germany). Survival curves were generated using Graphpad Prism v5 (GraphPad Software, Inc. USA). All tumors expressed ECI2 at varying intensity as punctate staining within the cytoplasm. Tumors were characterized according to the 3 categories of expression (Low, Medium, High, see Supplementary Figure 1) and correlated with overall survival (time from diagnosis to death / loss of follow up) and described using a Kaplan Meier curve (statistical analysis data are provided in Supplementary Table 5).

Gas-chromatography mass spectrometry

The aqueous fraction was derivitized for GC-MS using a two-step methoximation/silylation procedure [3]. ^{13}C-Serine (20µl, 1mM) and U-^{13}C-Glucose (20 µl, 1 mM) and Myristic acid d27 (10 µl, 1.5 mg/ml) were added to the samples as derivatization standards. The dried samples were methoximated using a solution of 20 mg/ml methoxyamine hydrochloride in anhydrous pyridine (20 µl) and incubated at 30°C for 90 min. Samples were then silylated by adding 80 µl MSTFA (with 1% TMCS) (Thermo) and incubating at 37°C for 30 min. 2-fluorobiphenyl in anhydrous pyridine (10 µl, 1 mM) was added to the samples as an injection standard and the samples were transferred to deactivated glass vial

inserts. GC-MS analysis was performed on an Agilent 7890 GC equipped with a 30 m DB5MS capillary column with a 10 m Duraguard column connected to an Agilent 5975 MSD operating under electron impact (EI) ionization (Agilent Technologies UK Ltd.). Samples were injected into deactivated splitless liners using an Agilent 7693 autosampler injector according to the method of Fiehn *et al*. [3]. Helium was the carrier gas. Metabolites were assigned using the Fiehn Library [3] and the deconvolution program AMDIS (Stein SE (1999) Journal of the American Society for Mass Spectrometry 10: 770–781. doi:10.1016/S1044-0305(99)00047-1.). MatLab scripts developed in-house were used to integrate metabolite peak areas for all samples [4].

^{1}H NMR spectroscopy of cell culture media

550 µL of culture medium, 50 µL of 11.6 mM sodium 4,4-dimethyl-4-silapentane-1-sulfonate (Onyx Scientific,(UK)), and deuterium oxide (D2O) as internal standard were mixed and transferred to 5 mm NMR tubes for spectroscopic analysis. Carr-Purcell-Meiboom-Gill (CPMG) 1H NMR spectra were acquired using a 14.1T Bruker AVANCE 600 spectrometer (600.1 MHz 1H frequency). All spectral acquisitions were made using a 5 mm TXI probehead (Bruker Biospin, Germany) at 300 Kelvin. The pulse sequence (RD-90°-(t-180°-t)n-AQ) was used and the fixed echo time, t, was set to 400µs, which yielded total spin echo time of 64ms. During the acquisition period (AQ, 2.73 s), the free induction decay (FID) was recorded into 64k data-points in the time domain, with a spectral width of 20ppm. Spectra were recorded as the sum of 128 transients following 8 dummy scans.

Lipidomics

Cell pellets were diluted in 700 µl PBS and mixed with 800 µl 1 N HCl:CH3OH 1:8 (v/v), 900 µl CHCl3 and 200µg/ml of 2,6-di-tert-butyl-4-methylphenol (BHT; Sigma Aldrich). After mixing for 5min in a rotary shaker and phase separation (by centrifugation at 17300 g, for 5 min at 4°C), the lower organic fraction was collected using a glass Pasteur pipette and evaporated using a Savant Speedvac spd111v (Thermo Fisher Scientific) at room temperature. The collected lipid pellets were stored at −20°C under argon. Just before mass spectrometry analysis, lipid pellets were reconstituted in running solution (CH_3OH:$CHCl_3$:NH_4OH; 90:10:1.25, v/v/v) depending on the amount of DNA of the original cell sample (1 µl diluent/1 µg DNA). Phospholipid species were analyzed using electrospray ionization tandem mass spectrometry (ESI-MS/MS) on a hybrid triple quadrupole/linear ion trap mass spectrometer (4000 QTRAP system; AB SCIEX) equipped with a TriVersa NanoMate (Advion Biosciences) robotic nanosource for automated sample injection and spraying. To quantify individual phospholipid species, the system was operated in multiple reaction monitoring (MRM) mode. MRM transitions were based on precursor ion or neutral loss scanning at collision energies set to 50 eV/45 eV, 35 eV, -35 eV and -60 eV for precursor 184 (phosphatidylcholine (PC)), neutral loss 141 (phosphatidylethanolamine (PE)), neutral loss 87 (phosphatidylserine (PS)) and precursor 241 (phosphatidylinositol (PI)), respectively. Lipid standards PC25:0, PC43:6, PE25:0, PE43:6, PI25:0, PI31:1, PI43:6, PS25:0, CER d18:1/17:0, PS31:1, PS37:4 (Avanti Polar Lipids) were added to each sample based on the amount of DNA. Individual MRM signals were averaged over a period of 3 min for each spectrum. Data were corrected for carbon isotope effects and chain length using in house-developed software (RALP 4.0). Phospholipid species displaying an intensity of at least 5 times the blank (consisting of only running solution) were considered.

Prepation of cell lysates

Chromatin immunoprecipitation was performed as described [5]. mRNA was collected for RNA-seq and RT-qPCR using illustra RNAspin Mini Kit, 50 preps, GE Healthcare kit (GE Healthcare, catalogue number: 25-0500-71), and reverse-transcribed with qScript cDNA Synthesis Kit (Quanta Biosciences, catalogue number: 95047-100). RT-qPCR was performed using SYBR-green reagent and primers used are listed in Supplementary table 3. For Western blotting (WB), cells were washed once with PBS and harvested into cell lysis buffer (10 mmol/L Tris-HCl, pH 8.0, 1mmol/L EDTA, 1% Triton X-100, 0.1% sodium-deoxycholate, 0.1% SDS, 140 mmol/L NaCl supplemented with Complete protease inhibitor mixture and Complete phosphatase inhibitor mixture; Roche), rotated for 15 minutes, sonicated with Bioruptor instrument and centrifuged 14,000g 10 minutes. Supernatans were collected and protein concentration determined using the BCA assay. Antibodies used for WB were: ECI2 (Sigma HPA022130), Actin (Cell Signaling Technology, CST, 5125), GAPDH (CST 2118), Cl-PARP (CST 9541), P62 (BD Biosciences 610833), LC3 (CST 2775), α-tubulin (CST 5346). The intensity of western blot signals were determined using Quantity One software (Bio-Rad).

Oil red O staining and lipid tox staining

For Oil Red O staining, cells were cultured in 6-well plates. At the point of harvesting, cells were fixed with 4% pFa for 20 minutes, washed with PBS, washed with 60% isopropyl alcohol and then Oil Red O working solution was added for 15–30 minutes (working solution was prepared by adding 4 parts of water + 3 parts of Oil Red O (Sigma), and the solution was allowed to rest for 15 minutes). After this, cells were washed with 60% isopropyl alcohol and PBS, and representative images were taken. In order to extract the Oil Red O stain,

1 mL of isopropyl alcohol was added and the plate was incubated on a tilting board 5 min, and after this the solution was centrifuged. Oil Red O signal was measured with spectrophotometer at 518 nM (isopropanol was used as blank).

For LipidTox staining, cells were plated into 384-plate in 12 technical replicates for each condition. At harvesting, cells were fixed with 4% formalin for 15 minutes, washed with PBS, and stained with the LipidTox stain. After this the signal was recorded with plate reader.

Analysis of the RNA-seq and re-analysis of ChIP-seq datasets

Tophat (v2.0.10) was used to align paired-end reads with bowtie (v2.1.0.0) to human genome version hg19. Cufflinks (v2.1.1) was used to build transcript assemblies and to retrieve differentially expressed genes according to the standard protocol [6]. Differentially expressed transcripts were compared to scrambled siRNA in each cell line and overlapped to achieve a consensus of the affected transcripts upon treatment with two different siRNAs. RNA-seq data has been made available: http://www.ncbi.nlm.nih.gov/geo/query/acc.cgi?token=wxyd ygwubzcxxax&acc=GSE75035 for editor / reviewers, and will be made publicly available once the manuscript has been accepted. The processed files are compressed folders containing multiple output files from CuffDiff runs estimating differentially expressed transcripts between the indicated ECI2 siRNA treated cells versus cells treated with Scrambled siRNAs (see Trapnell *et al.*, 2012 for more info [6]).

Consensus AR binding in several castration resistant prostate cancer tissues [7], was retrieved using lift-over tool.

Analysis of mayo cohort samples for met- and prostate cancer specific mortality-free survival

The Mayo cohort was designed as a case-cohort study as described previously [8] for a group of men in matched triples of metastatic progression ($N = 213$), biochemical recurrence after radical prostatectomy ($N = 213$) and patients with no evidence of disease ($N = 213$). 212 patients with metastatic progression and 333 patients without metastatic progression were evaluated. Sample selection, RNA extraction, and microarray hybridization were performed in a Clinical Laboratory Amendments (CLIA)-certified laboratory facility (GenomeDx Biosciences, San Diego, CA, USA) as described [9, 10]. After quality control was performed using Affymetrix Power Tools packages, probeset normalization and summarization were performed using the Single Channel Array Normalization (SCAN) algorithm [11]. Gene expression was summarized using the Affymetrix Core level summaries for annotated genes. Statistical analyses were performed in R, version 3.2.0 and all statistical tests were two-sided using a 5% significance level. Kaplan-Meier and survival analysis using Cox's regression model (survival 2.38-1) were performed and significance between groups of the Kaplan-Meier curve were assessed using the log-ranked test. Groups were defined as high and low expression based on median cut-points.

REFERENCES

1. Morrison C, Cheney R, Johnson CS, Smith G, Mohler JL. Central quadrant procurement of radical prostatectomy specimens. The Prostate. 2009; 69:770–773.
2. Livak KJ, Schmittgen TD. Analysis of relative gene expression data using real-time quantitative PCR and the 2(-Delta Delta C(T)) Method. Methods. 2001; 25:402–408.
3. Kind T, Wohlgemuth G, Lee do Y, Lu Y, Palazoglu M, Shahbaz S, Fiehn O. FiehnLib: mass spectral and retention index libraries for metabolomics based on quadrupole and time-of-flight gas chromatography/mass spectrometry. Analytical chemistry. 2009; 81:10038–10048.
4. Behrends V, Tredwell GD, Bundy JG. A software complement to AMDIS for processing GC-MS metabolomic data. Analytical biochemistry. 2011; 415:206–208.
5. Massie CE, Lynch A, Ramos-Montoya A, Boren J, Stark R, Fazli L, Warren A, Scott H, Madhu B, Sharma N, Bon H, Zecchini V, Smith DM, et al. The androgen receptor fuels prostate cancer by regulating central metabolism and biosynthesis. The EMBO journal. 2011; 30:2719–2733.
6. Trapnell C, Roberts A, Goff L, Pertea G, Kim D, Kelley DR, Pimentel H, Salzberg SL, Rinn JL, Pachter L. Differential gene and transcript expression analysis of RNA-seq experiments with TopHat and Cufflinks. Nature protocols. 2012; 7:562–578.
7. Sharma NL, Massie CE, Ramos-Montoya A, Zecchini V, Scott HE, Lamb AD, MacArthur S, Stark R, Warren AY, Mills IG, Neal DE. The androgen receptor induces a distinct transcriptional program in castration-resistant prostate cancer in man. Cancer cell. 2013; 23:35–47.
8. Nakagawa T, Kollmeyer TM, Morlan BW, Anderson SK, Bergstralh EJ, Davis BJ, Asmann YW, Klee GG, Ballman KV, Jenkins RB. A tissue biomarker panel predicting systemic progression after PSA recurrence post-definitive prostate cancer therapy. PloS one. 2008; 3:e2318.
9. Erho N, Crisan A, Vergara IA, Mitra AP, Ghadessi M, Buerki C, Bergstralh EJ, Kollmeyer T, Fink S, Haddad Z, Zimmermann B, Sierocinski T, Ballman KV, et al. Discovery and validation of a prostate cancer genomic classifier that predicts early metastasis following radical prostatectomy. PloS one. 2013; 8:e66855.
10. Ross AE, Johnson MH, Yousefi K, Davicioni E, Netto GJ, Marchionni L, Fedor HL, Glavaris S, Choeurng V, Buerki C, Erho N, Lam LL, Humphreys EB, et al. Tissue-

based Genomics Augments Post-prostatectomy Risk Stratification in a Natural History Cohort of Intermediate- and High-Risk Men. European urology. 2015.

11. Piccolo SR, Sun Y, Campbell JD, Lenburg ME, Bild AH, Johnson WE. A single-sample microarray normalization method to facilitate personalized-medicine workflows. Genomics. 2012; 100:337–344.

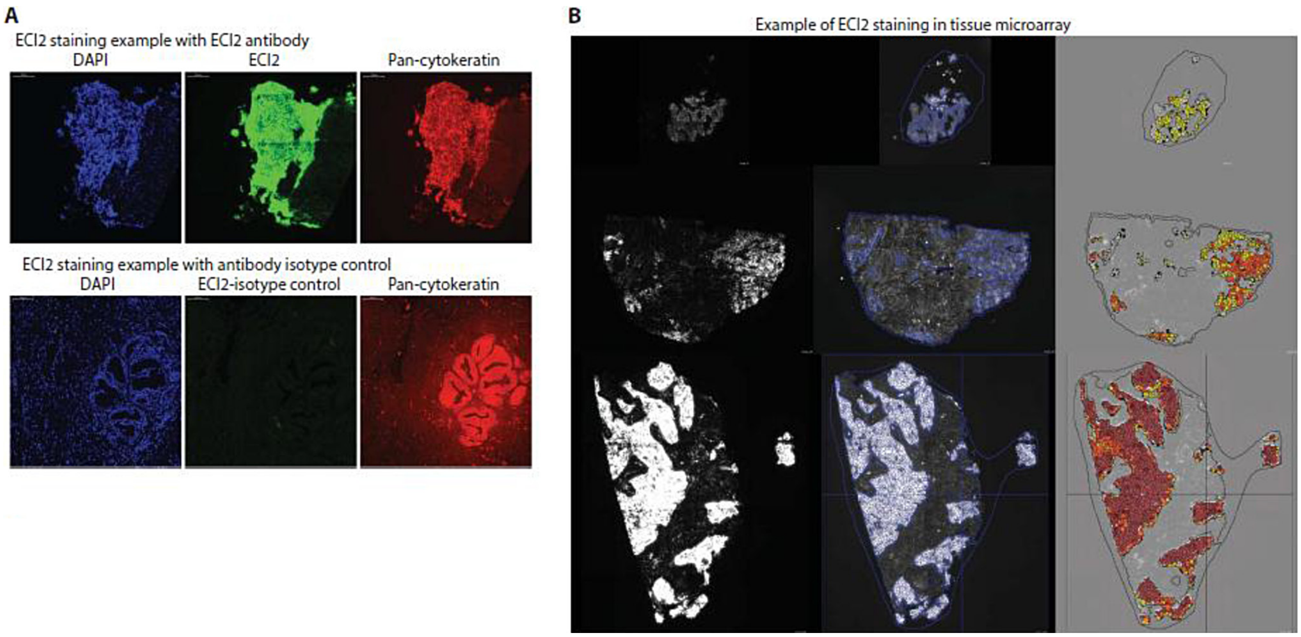

Supplementary Figure 1: ECI2 staining in prostate cancer TMA. (**A**) Evaluation of ECI2-antibody. Prostate cancer samples were stained as indicated in the figure to assess the specificity of the ECI2 antibody. (**B**) Representative images used to quantitate ECI2 expression in prostate cancer patient samples. The first image on the left is a composite of immune-staining. The image in the middle shows the original ECI2 fluorescence image next to the Definiens cell identification (Defined by DAPI and pan CK). The image on the right shows the classification by Definiens of each epithelial cell (yellow–Low; amber–medium; red–high) for immune-staining. Running top to bottom are examples of low, medium and high grade immune-staining. The TMA was set up using needle core biopsies and tissue/tumor content has been confirmed by 2 independent pathologists. The staining for ECI2, and other markers were performed with an automated Definiens system. This system requires the operator to define an algorithm (in this case, all epithelial cells (Dapi^{+ve} and pan-cytokeratin^{+ve}). The Definiens system then determines the intensity of staining in each tumor cell automatically and produces a score which was used to stratify the patients.

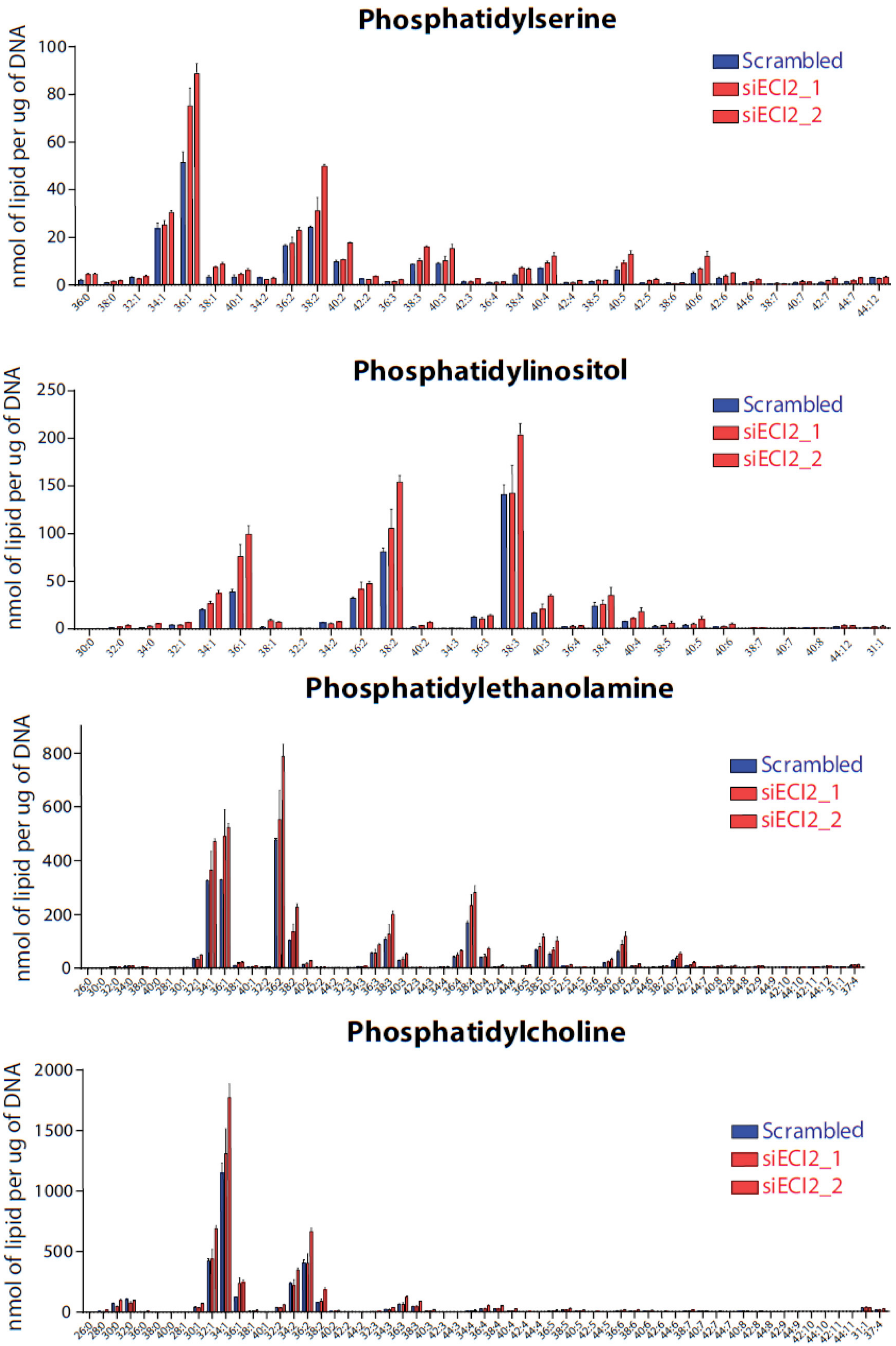

Supplementary Figure 2: Lipidomic profiling after 72 hours of ECI2 knockdown in LNCaP cells. The levels of intracellular phosphatidylserine, phosphatidylinositol, phosphatidylethanolamine and phosphatidylcholine were determined using mass-spectrometry after 72 hours of ECI2 knockdown. Data shown are an average (with SEM) of three biological replicates.

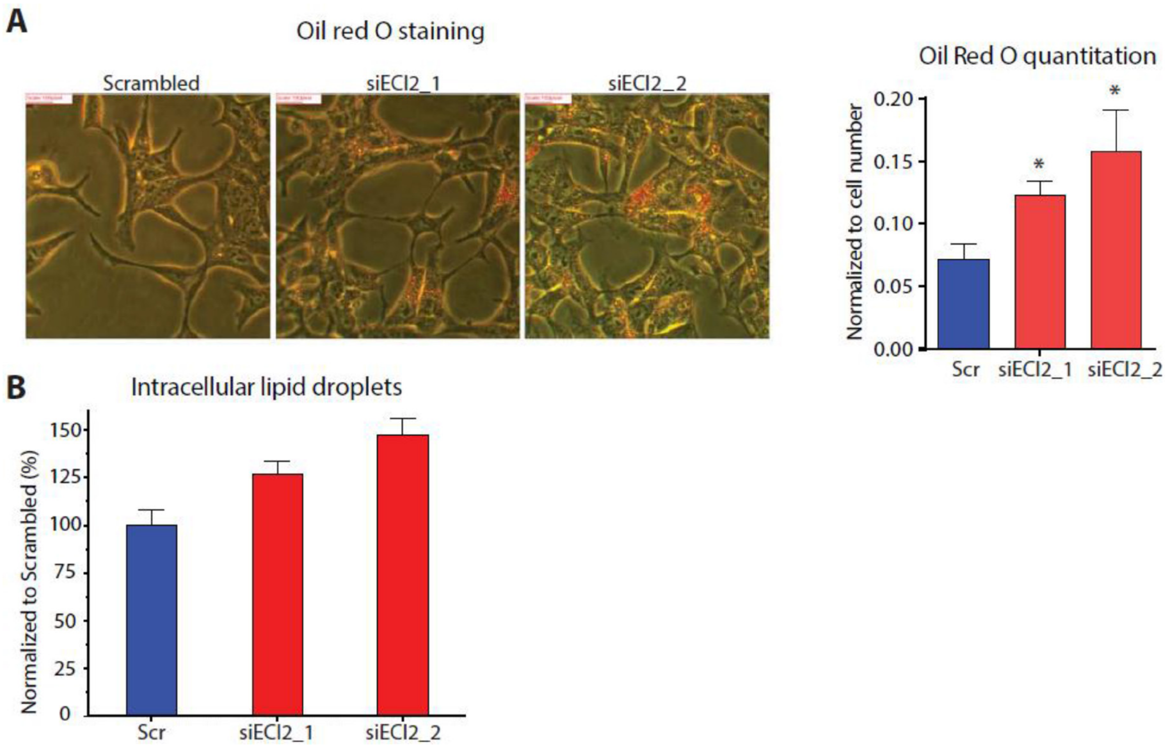

Supplementary Figure 3: ECI2 knockdown induces lipid accumulation into LNCaP cells. (**A**) Cells were stained with Oil Red O as detailed in materials and methods. Data shown are an average (with SEM) of four biological replicates. The significance was evaluated using paired samples Student's *t*-test, *< 0.05. (**B**) Lipid droplet quantitation after 72 hours of ECI2 knockdown. Cells were stained with LipidTox stain as detailed in materials and methods. The data shown is representative of two biological replicates, and error bars are standard deviation of 12 technical replicates. Scrambled sample was set to 100%, and siECI2-samples were normalized to this.

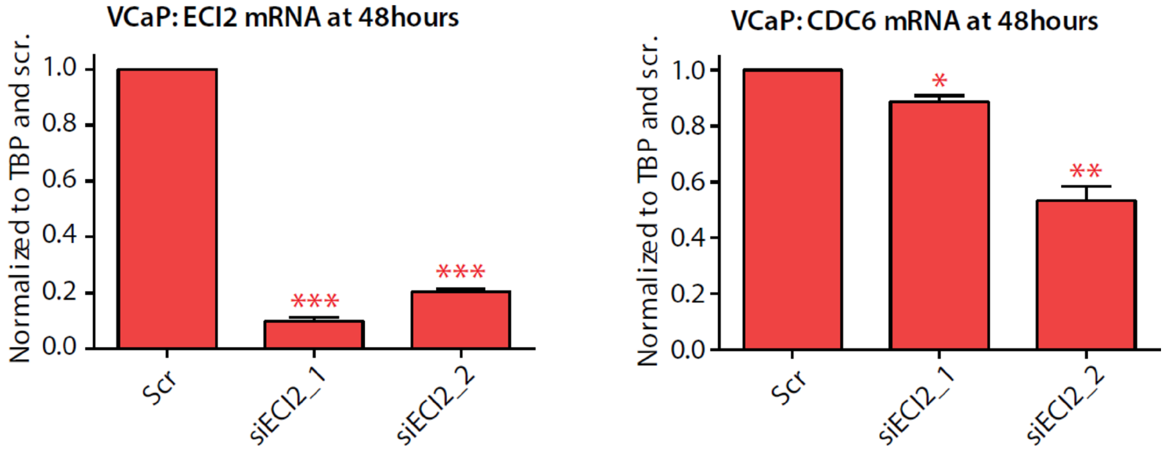

Supplementary Figure 4: CDC6 expression is decreased after ECI2 knockdown in VCaP cells. ECI2 was knocked down using siRNA for 48 hours and RNA was collected and used for RT-qPCR. The data shown are an average of four biological replicates with SEM, and statistical analysis was performed with Student's *t* test, *< 0.05, **< 0.01, ***< 0.001.

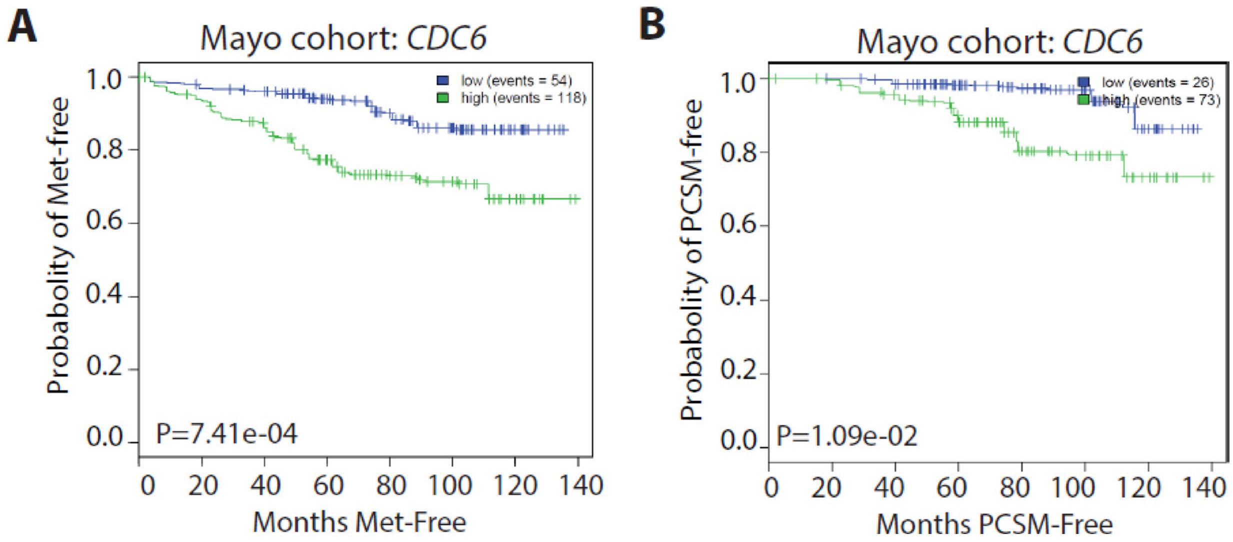

Supplementary Figure 5: Increased *CDC6* expression is associated with aggressive prostate cancer. Samples were split by the median expression of *CDC6* into groups of low and high expression. (**A**) Metastasis (Met)-free survival for *CDC6* low and high expression groups of samples from the Mayo case–cohort data set [9]. (**B**) Prostate cancer specific mortality (PCSM)-free survival for CDC6 low and high expression groups of samples from the Mayo case–cohort data set [9].

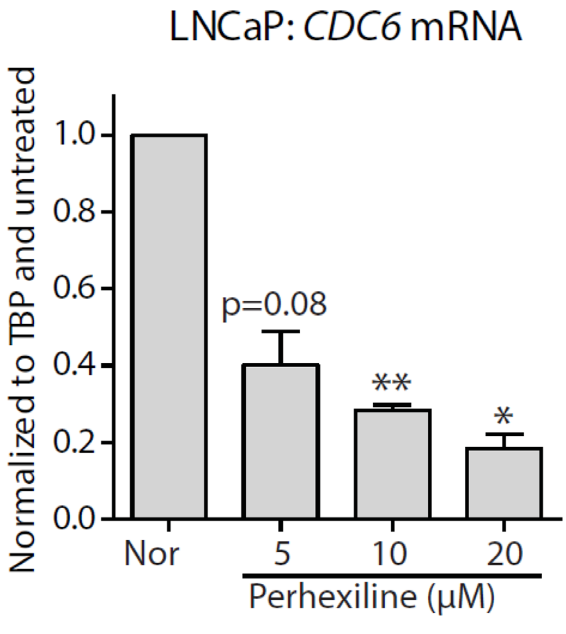

Supplementary Figure 6: Lipid degradation inhibitor perhexiline decreases the expression of CDC6 in prostate cancer cells. LNCaP cells were treated with perhexiline for 4 hours and mRNA was collected. The values were first normalized to a house-keeping gene (TBP) and then to untreated sample. Data shown is an average of three biological replicates with SEM. Statistical analysis was performed with Student's *t* test, *< 0.05, **< 0.01.

Supplementary Table 1: Pathway enrichment analysis after ECI2 knockdown (up-regulated genes top, down-regulated genes below)

Term	*p*-Value	Genes
hsa04115:p53 signaling pathway	0.06512373050130565	CDKN1A, SESN2
hsa05220:Chronic myeloid leukemia	0.0716301025027497	CDKN1A, HDAC1

Term	*p*-Value	Genes
hsa04110:Cell cycle	8.104830917220123E-7	CDK1, CDC6, MAD2L1, CCND3, CDKN2C, CDC25A, WEE1, CDC25B
hsa04914:Progesterone-mediated oocyte maturation	5.284021199068409E-4	CDK1, MAD2L1, PIK3CB, CDC25A, CDC25B
hsa04630:Jak-STAT signaling pathway	0.03146195304502031	CCND3, PIK3CB, CBL, SOCS5
hsa04540:Gap junction	0.06042602445198117	CDK1, TUBA1A, TUBA1B

Pathway enrichment analysis was done based on supplementary table "ECI2-dependent transcriptome in LNCaP and RWPE-1 cells", and this data is provided as a separate table.
Database for Annotation, Visualization and Integrated Discovery (DAVID) was used to evaluate what pathways are affected in LNCaP cells after ECI2 knockdown.

Supplementary Table 2: Clinicopathological characteristics of the patients used to evaluate ECI2 expression in mRNA level

Clinicopathological characteristics	
Age	
Median	61
Interquartile range	54–66
PSA	
Median	7.2
Interquartile range	(4.0–10.9)
Gleason score	
6	5
7a	6
7b	5
8	2
9	2
T stage	
2c	14
3a	2
3b	2
4	2
LN status	
N0	12
N1	0
Nx	8

Supplementary Table 3: Probes and primers used in this study

	TaqMan probe (used for patient sample profiling)
ECI2	Hs00196146_m1
18s	Hs99999901_s1
GAPDH	Hs99999905_m1
TBP	Hs99999910_m1

	Primer-forward (used for cell line samples)	Primer-reverse (used for cell line samples)
ECI2-ChIP primers	CCAGGCTCCACCTGACTAGA	ATGGGCTGAGGTTGTTTGTC
Negative site-ChIP	CAGTGGCCATGAGTTTTGTTTG	AACCAATCCAACTGCATTATACACA
ECI2	CCAGGCTCCACCTGACTAGA	GGCCACTGAAGGACCTTGTA
Actin	TGGGACGACATGGAGAAAAT	AGAGGCGTACAGGGATAGCA
TBP	GCCAGCTTCGGAGAGTTCTG	GCACGAAGTGCAATGGTCTTT
CDKN1A	CAGATTTGTGGCTCACTTCGTG	CCTGCGTTGGTGCGCT
CDK1	GGTTCCTAGTACTGCAATTCG	TTTGCCAGAAATTCGTTTGG
CDC6	GGCCAGGATGTATTGTACAC	GGCCCGAATGTGTAAAGC
CDC25A	GTGGCCTCCCTCGTATTCAT	AGTTGCTGGGAAACATCAGG
AURKB	CAGTGGGACACCCGACATC	GTACACGTTTCCAAACTTGCC

Supplementary Table 4: Tissue microarray demographics

Number of Patients		144
Average Age		72.67
% Gleason Score	≤ 6	40
	7	16
	≥ 8	26
	unknown	18
% T Stage	T1	7
	T2	24
	T3	39
	T4	24
	unknown	6
% M Stage	M0	39
	M1	31
	Mx	26
	unknown	4
Status	Alive	70
	Dead	74

Supplementary Table 5: Tissue microarray statistics table for the survival curves between the high and low/medium expression of ECI2

**Comparison of Survival Curves**	
Log-rank (Mantel-Cox) Test	
Chi square	6.896
df	1
P value	0.0086
P value summary	**
Are the survival curves sig different?	Yes
Gehan-Breslow-Wilcoxon Test	
Chi square	6.067
df	1
P value	0.0138
P value summary	*
Are the survival curves sig different?	Yes
Median survival	
Low/Med	115.0
High	77.00
Ratio	1.494
95% CI of ratio	0.8861 to 2.101
Hazard Ratio	
Ratio	0.5311
95% CI of ratio	0.3312 to 0.8517

Supplementary Table 6: ECI2-dependent transcriptome in LNCaP and RWPE-1 cells. See Supplementary_Table_6
